# Coexistence of persistent left superior vena cava which is drained directly to left atrium and aortic stenosis with low gradient and low ejection fraction

**DOI:** 10.1186/1749-8090-8-S1-P20

**Published:** 2013-09-11

**Authors:** O Tetik, Y Besir, S Surer, O Rodoplu

**Affiliations:** 1 Department of Cardiovascular Surgery, Manisa Celal Bayar University School of Medicine, Manisa, Turkey; 2 Department of Cardiovascular Surgery, Ataturk Training and Research Hospital, Izmir, Turkey; 3 Department of Cardiovascular Surgery, Yuksek Ihtisas Training and Research Hospital, Bursa, Turkey

## Background

Persistent left superior draining into the left atrium is very rare. In this case report we are presenting a patient with persistent left superior vena cava draining to left atrium which was probably causing left-right shunt.

## Methods

46-year-old male patient was hospitalized because of shortness of breath. The patient's general condition was very bad and he was confined to a wheelchair. The patient was in New York Heart Association Functional Classification (NYHA FC) Class IV. Severe aortic stenosis, EF 20%, Left ventricular end-systolic diameter 65 mm, end-diastolic diameter 50mm, global hypokinesia, gradient 51/13 mm Hg, normal mitral valve, pulmonary hypertension (75 / 24mmHg) were determined on transthoracic echocardiography. Basal gradient increased from 51/13mm Hg to 72/40 mmHg on dobutamine stress echocardiography.

## Results

Intraoperativelly, persistent left superior vena cava draining to left atrium was seen. Oxygen saturation was 59% in the left subclavian vein and 83 in the left innominate vein was causing left-right shunt due to severe aortic stenosis. Pulmonary arterial pressure decreased after Persistent left superior vena clampage and then the vein was ligated. Aortotomy was performed. Severe aortic stenosis on leaflet level and calcification were seen. The aortic valve was replaced with no 21 Sorin bileaflet mechanical valve. The Patient was followed-up for two days in intensive care unit and was discharged on postoperative seventh day. Significant improvement in patient’s transthoracic echocardiography findings EF 40% and pulmonary artery pressure 30/14 mm Hg) and physical condition were seen on Postoperative six month outpatient control.

## Conclusions

Our patient also had persistent left superior vena cava draining directly to left atrium. Probably due to severe aortic stenosis it was causing a shunt from left to right because severe pulmonary hypertension and right ventricular dilatation declined shortly after ligation of the vein.

